# Zinc phthalocyanines as light harvesters for SnO_2_-based solar cells: a case study

**DOI:** 10.1038/s41598-020-58310-1

**Published:** 2020-01-24

**Authors:** Riccardo Milan, Gurpreet Singh Selopal, Marco Cavazzini, Simonetta Orlandi, Rita Boaretto, Stefano Caramori, Isabella Concina, Gianluca Pozzi

**Affiliations:** 1 Department of Information Engineering, University of Brescia, Via Valotti, 9 – 25131 Brescia Italy; 2 CNR-INO SENSOR Laboratory, via Branze, 45 – 25123 Brescia Italy; 3 Institute of Fundamental and Frontier Sciences, University of Electronic Science and Technology of China, Chengdu, 610054 P. R. China; 4 Institut National de la Recherche Scientifique, Centre Énergie, Matériaux et Télécommunications, 1650 Boul. Lionel Boulet, Varennes, QC J3X 1S2 Canada; 5 Institute of Molecular Science and Technology, ISTM-CNR, Via Golgi, 19, 20133 Milano Italy; 6 Department of Chemical and Pharmaceutical Sciences, University of Ferrara, Via Borsari, 46, 44121 Ferrara Italy; 7 Division of Materials Science, Department of Engineering Sciences and Mathematics, Luleå University of Technology, 971 87 Luleå, Sweden

**Keywords:** Solar cells, Energy

## Abstract

SnO_2_ nanoparticles have been synthesized and used as electron transport material (ETM) in dye sensitized solar cells (DSSCs), featuring two peripherally substituted push-pull zinc phthalocyanines (ZnPcs) bearing electron donating diphenylamine substituents and carboxylic acid anchoring groups as light harvesters. These complexes were designed on the base of previous computational studies suggesting that the integration of secondary amines as donor groups in the structure of unsymmetrical ZnPcs might enhance photovoltaics performances of DSSCs. In the case of TiO_2_-based devices, this hypothesis has been recently questioned by experimental results. Herein we show that the same holds for SnO_2_, despite the optimal matching of the optoelectronic characteristics of the synthesized nanoparticles and diphenylamino-substituted ZnPcs, thus confirming that other parameters heavily affect the solar cells performances and should be carefully taken into account when designing materials for photovoltaic applications.

## Introduction

In spite of the loss of fame following the advent of perovskite solar cells, dye sensitized solar cells (DSSCs), which can be manufactured from eco-friendly, low-cost materials, still deserve attention due to the careful materials engineering and coupling they are the object of, asking scientists of different fields to join efforts to shine light on basic phenomena. Major efforts in DSSCs are still devoted to cell components engineering^1,2^, including the seek for wide band gap semiconducting metal oxides (MOx) as electron transport materials (ETM) alternative to TiO_2_^1–6^, and to the development of new photosensitizers enabling the exploitation of a wider portion of the solar spectrum^7^. Investigations should be then focused not only to enhance the overall functional performances of the solar energy converting devices, which are so far limited, but especially to unveil fundamental phenomena and processes.

In the field of ETMs to be used as photoanodes in DSSCs, SnO_2_ is an interesting candidate, assuming that a material-by-design approach is adopted in the preparation of nanostructures featuring proper optoelectronic characteristics, such as light management, charge transport and energy alignment with the chosen sensitizer. Indeed, over the years several attempts to optimize the aforementioned parameters by means of material shaping^8–13^, capping/mixture with other oxides^14–19^, or doping^20,21^ have been reported. Most of these studies dealt with the use of SnO_2_ nanoparticles (SnO_2_ NPs) of size spanning from about 6 to 140 nm, and were focused on understanding the role of light-matter interaction and charge transport through tin dioxide^14,15,17,20–23^.

In the field of photosensitizers, phthalocyanines (Pcs) play a prominent role due to their excellent photo- and electrochemical stability and unique light harvesting capability in the red/NIR spectral regions, allowing the exploitation of a relevant portion of the solar photon flux that cannot be captured by other dyes^24^. Such properties can be finely modulated by proper synthetic modifications that are relatively simple to perform^25,26^. The optoelectronic properties of Pcs can indeed be effectively tuned by varying the organic substituents on the macrocyclic core, as this approach is successful in the modulation of the HOMO-LUMO energy levels and of the electron density distribution. Metallation of the Pc core, in particular coordination of Zn(II) that enables the existence of long-lived singlet excited states, is another strategy that proved of great utility for DSSCs applications^24^. Because of this remarkable versatility, Pcs are appealing candidates as light harvesters in DSSCs, as witnessed by an impressive number of literature examples dealing with their use in combination with TiO_2_ photoanodes^27–32^. These studies also highlighted some issues seriously affecting the application of Pcs in TiO_2_-based DSSCs, among which their aggregation on the titania surface^33^, leading to the formation of never-ending dye layers on the ETM, jeopardizing the device functional performances due to enhanced exciton recombination^34^. Unsymmetrically substituted Pcs bearing bulky, electron-donating groups on three of the four isoindole subunits which constitute the Pc macrocycle, and one or more electron-withdrawing groups able to interact with MOx on the forth one have been designed to alleviate this issue^27,28^. However, the addition of co-adsorbing agents (e.g. deoxycholic acid), competing with the sensitizer for anchoring sites on the TiO_2_ surface and hindering interactions between Pc units, remains instrumental even with this favorable molecular pattern^31,35^.

With these limitations in mind and following our continuous efforts to develop both ETMs and light harvesters for DSSCs, with particular focus on SnO_2_ and ZnO as alternative materials for photoanodes^36–38^, and on dyes capable of efficiently replacing the most commonly used Ru(II) complexes^39–42^, we herein investigate the unprecedented use of Zn(II) complexes of unsymmetrically substituted push-pull Pcs (ZnPcs) as photosensitizers in DSSCs featuring SnO_2_ NPs as ETM.

## Results and Discussion

**Figure 1:**
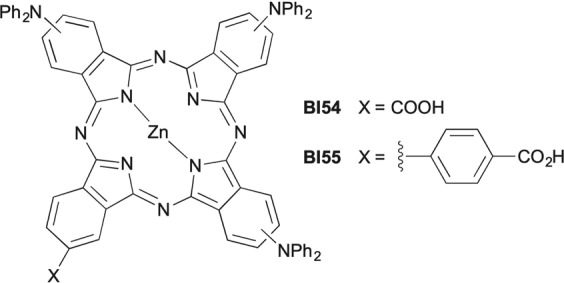
Peripherally substituted ZnPcs BI55 and BI54 bearing diphenylamino donors.

The most successful ZnPcs sensitizers reported so far are characterized by the concurrent presence of electron-rich aryloxy groups –OAr rotated with respect to the macrocycle plane and -COOH binding groups, according to a structural pattern essentially developed by a trial and error approach^24,27,28^. Computational techniques were also applied to support a rational molecular design of unsymmetrical push-pull ZnPcs, strongly advocating the use of -NH_2_, -NHR, -NR_2_, NHPh or -NPh_2_ donor groups instead of –OAr to improve DSSC functional performances^43–45^. In order to test the validity of these predictions, we recently made available complex **BI54** (Fig. 1), the first example of unsymmetrical ZnPc bearing -NPh_2_ donor groups.

While the measured optoelectronic parameters of **BI54** were in good agreement with computational models and potentially useful for DSSC applications, the photovoltaic performance of the dye when TiO_2_ NPs were used as ETM disproved theoretical predictions^46^. This inconsistency was ascribed, among others, to the problematic adsorption of **BI54** on TiO_2_ NPs and to the possible shift of the CB of TiO_2_ leading to a decreased charge injection efficiency from the excited state of the sensitizer. It was thus interesting to investigate the photovoltaic behavior of **BI54** in combination with a different ETM. On the other hand, the binding mode of unsymmetrical ZnPcs on MOx and their photovoltaic behavior are strongly influenced by the interposition of a linker between the Pc core and the anchoring group^33,47–49^. We thus synthesized **BI55** (Fig. 1, see Supplementary Materials for details), an analog of **BI54** where the Pc core and the –COOH anchoring group are connected by an aryl ring, and tested the two ZnPcs in parallel as photosensitizers in DSSCs fabricated with SnO_2_ NPs as ETM.

The energy levels of the HOMO-LUMO orbitals of **BI55** were assessed by electrochemical measurements combined with the analysis of the optical properties (Figures S1 and Table S1 in the Supplementary Materials). With HOMO and LUMO positioned at about −5.5 eV and −3.8 eV, respectively, and a typical UV-Vis absorption spectrum characterized by strong bands centered at 353 nm (Soret band) and 718 nm (Q band), the new dye satisfies the thermodynamic requirements for an effective use as light harvester in DSSCs, analogously to **BI54**^46^.

**Figure 2:**
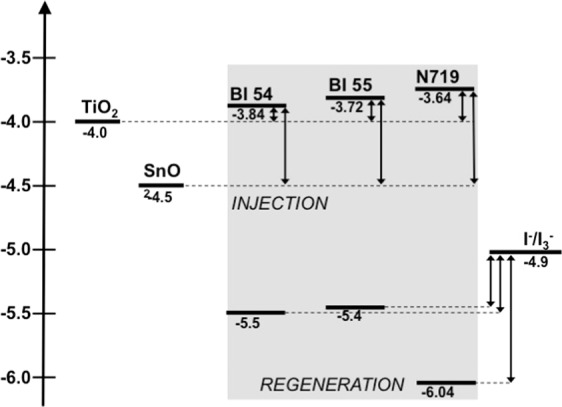
Energy band alignment of TiO_2_, SnO_2_ and BI54, BI55 dyes. Commercial Ru(II) based dye N719 is reported for comparison purposes. Energy position of HOMO and LUMO of BI55 and BI54 have been obtained by electrochemical and optical analyses as described in the text.

Thanks to a favorable band alignment (sketched in Fig. 2), the use of SnO_2_ should provide for a better driving force as for electron injection than that with TiO_2_, given that the nanostructured MOx is properly designed for photogenerated charge transport and light scattering. SnO_2_ is also expected to offer enhanced stability under UV exposure as compared with TiO_2_ thanks to the wider band gap^50^. Finally, SnO_2_ is fit for coupling with NIR absorbing molecules, thus appearing the ideal candidate to be tested with both ZnPcs.

**Figure 3:**
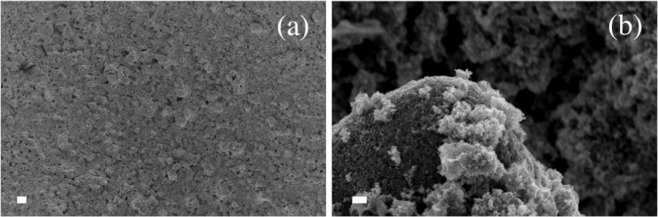
SEM images of SnO_2_ nanoparticles based photoanodes. Scale bar: (a) 20 μm; (b) 200 nm.

SnO_2_ nanoparticles used as ETM were synthesized through a wet chemical approach using methanol as a solvent. In order to check the complete removal of methanol, as well as any residual surface hydroxyl groups, Fourier transform infrared spectroscopy was applied. The spectra pertaining to the material after the drying procedure in oven and to the metal oxide after the calcination are reported in the Supplementary Materials (Figure S2). SEM analysis of SnO_2_ photoanodes (Fig. 3) evidences a homogenous deposition of the scattering layer over a wide area (Fig. 3a) and shows that the metal oxide is constituted of uniform spherical nanoparticles whose size is about 20 nm (Fig. 3b), as previously verified by XRD^36^.

**Figure 4:**
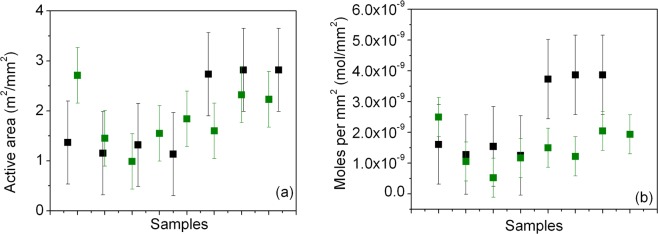
(**a**) Calculation of the active area per mm^2^ featured by the SnO_2_ (black markers) and TiO_2_ (green markers) photoanodes. (**b**) Dye loading for the photoelectrodes based on SnO_2_ (black markers) and TiO_2_ (green markers) NPs.

Estimation of the active area per mm^2^ of the SnO_2_ photoelectrode has been performed by using the commercial Ru(II) based dye N719 as a molecular probe, considering a molecular area as high as 1.6 nm^2^ for the dye^51^ and is reported in Fig. 4a, together with the data pertaining to TiO_2_ photoelectrode, used as a benchmark.

An average value of about 1.25 m^2^/mm^2^ has been calculated for the SnO_2_ NPs (TiO_2_ photoanodes, composed of both transparent and scattering layers, i.e. with particles featuring diameters from 20 nm to 150–250 nm on average, featured a value as high as 1.84 m^2^/mm^2^). Active area is a critical parameter since it relates to dye loading capability, hence to the current that can be photogenerated in the solar energy converting device. Dye loading has been also evaluated by detaching the adsorbed dye N719 and found be about 2.45 × 10^−9^ mol/mm^2^ for SnO_2_ photoanodes and 1.91 × 10^−9^ mol/mm^2^ for TiO_2_-based photoelectrodes (Fig. 4b). This is a relevant finding: it has indeed been previously reported a significantly lower dye loading for SnO_2_, ascribed to the more acidic nature of the SnO_2_ surface (anatase TiO_2_ presents the isoelectric point at pH 6.2, while for SnO_2_ it is at pH 4–5)^52^. Usually the lower photocurrent featured by SnO_2_-based DSSCs is then attributed to this difference in dye loading. However, in the present study, SnO_2_ shows a dye uptake capability slightly higher than TiO_2_ and, as we will see, this is reflected in the short circuit photocurrent density of the devices exploiting the two oxides as ETMs.

The functional parameters of various DSSCs based on SnO_2_ as ETM and dyes **BI54** or **BI55** as light harvester are reported in Table 1, together with data obtained using the commercially available dye N719.

**Table 1:** Functional parameters recorded for the dye sensitized solar cells presented in this work.

Entry No.	ETM/Dye	Additive	J_*SC*_ (mA/cm^2^)	V_*OC*_ (V)	FF (%)	PCE (%)
1	SnO_2_/N719	none	11.44	0.51	49	2.87
2	TiO_2_/N719	none	11.61	0.74	63	5.38
3	SnO_2_/BI54	none	0.355	0.07	19	0.005
4	SnO_2_/BI54	DCA	2.605	0.18	27	0.13
5	SnO_2_/BI54	DCA	2.38	327	41	0.32
6	SnO_2_/BI55	DCA	1.11	260	38	0.11
7	TiO_2_/BI54	DCA	1.376	0.54	58	0.43

As previously mentioned, ZnPcs are usually subjected to aggregation due to π-π stacking, which leads to the formation of adsorbed multi-layers onto the MOx surface, detrimental for functional performances. We have recently demonstrated^46^ that also **BI54** suffers of this issue on TiO_2_ photoelectrodes and we verified the same in case of SnO_2_. Indeed, solar converting device sensitized with dye **BI54** in the absence of co-adsorbing agents show very low functional performances (Entry 3 in Table 1 and Figure S1 in the Supplementary Materials). Deoxycholic acid (DCA) was thus added to the sensitizing mixture (50 mM), which resulted in enhancing all the functional parameters: J_*SC*_ in increased of more than seven times, V_*OC*_ about 2.5 times and an increase of 26 times is recorded as for the photoconversion efficiency (entry 4 in Table 1). Thus, the addition of co-adsorbing agents acting as molecular spacers is confirmed as critical in order to effectively keep the dye molecules separated on the MOx surface and to avoid the formation of never-ending layers atop the ETM surface.

In order to assess the capability of SnO_2_ NPs as ETM in DSSCs with respect to the more commonly used TiO_2_, the behavior of devices with photoanodes sensitized with two different dyes, N719 and **BI54**, was investigated.

**Figure 5:**
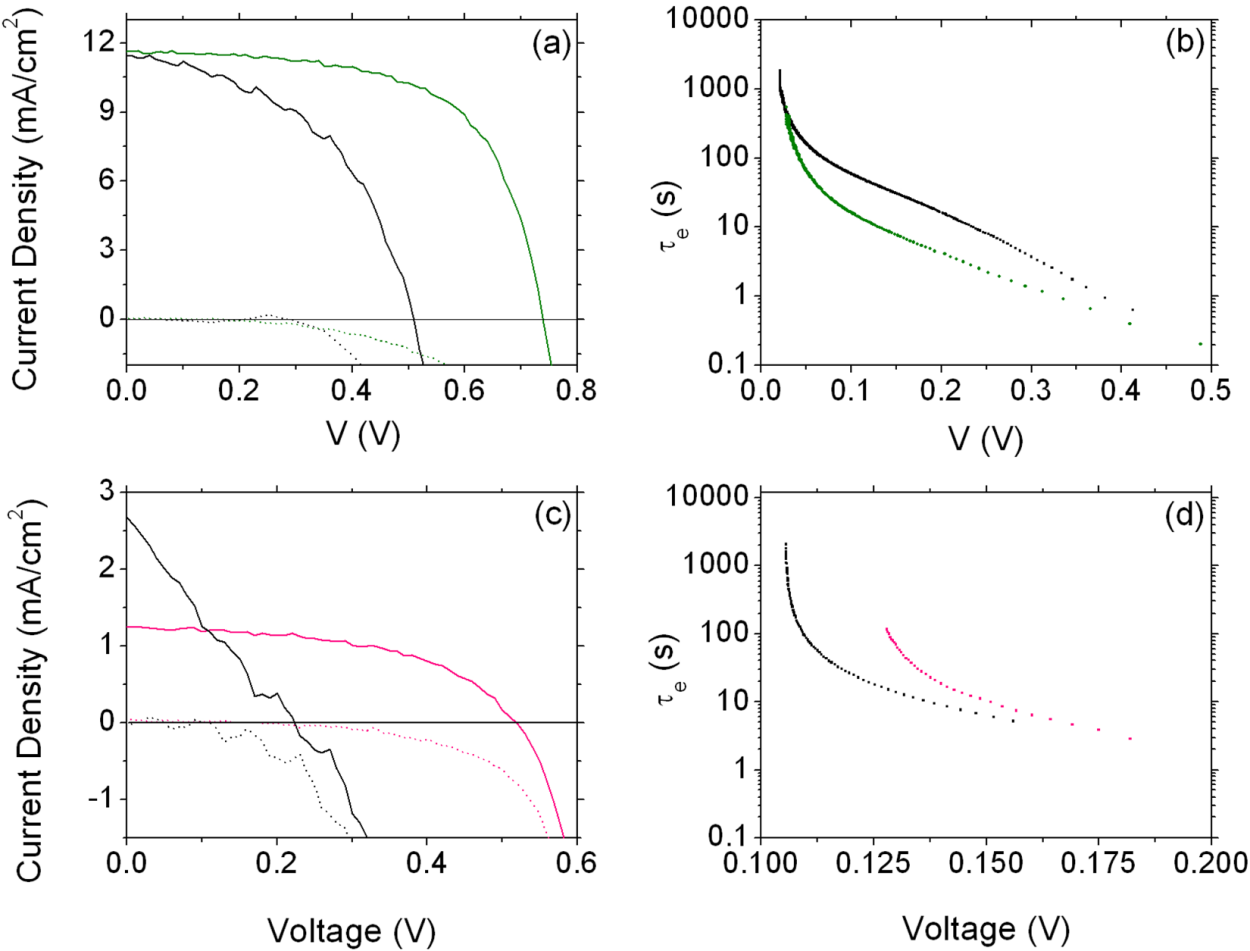
Comparison of the performance of TiO_2_ and SnO_2_ as ETM for DSSCs. (**a**) and (**c**) = *J-V* characteristics under simulated solar light (solid lines) and in dark (dashed lines); (**b**) and (**d**) = electron lifetime. (**a**) and (**b**): N719 is used as light harvester. (**c**) and (**d**): BI54 is used as light harvester. Black line: SnO_2_; green and pink lines: TiO_2_.

Figure 5a shows the *J-V* characteristics in dark and under simulated solar light of DSSCs sensitized with N719, where the ETM is either TiO_2_ (green lines) or SnO_2_ (black lines). Functional parameters are reported in Table 1, entries 1 and 2).

When the commercial Ru-based dye N719 is used as light harvester, a higher V_*OC*_ value is recorded for the TiO_2_-based device (740 mV) compared with the solar cell working with SnO_2_ as ETM (510 mV), as expected, while comparable short circuit current is observed (11.61 vs. 11.44 mA/cm^2^ for TiO_2_ and SnO_2_ photoanodes, respectively). Electron lifetime (Fig. 5b) is found to be slightly longer for the cell based on SnO_2_.

It is worth noting that the performances of the present bare (i.e. without any surface treatment aimed at reducing back recombination between the MOx CB and the electrolyte) SnO_2_-based DSSCs sensitized with N719 are much higher than those previously reported for other bare SnO_2_ photoanodes, as it can be seen in Table 2.

**Table 2:** Functional parameters of SnO_2_-based DSSCs reported in literature. ^‡^ Sizes inferred by SEM images reported in the supporting information of ref. ^20^

Photoanode	Notes	J_*SC*_ (mA/cm^2^)	V_*OC*_ (mV)	FF (%)	PCE (%)	Reference
Bare SnO_2_	20 nm NPs	11.44	510	49	2.87	This work
Bare SnO_2_	20 nm NPs SnO2 blocking layer on FTO glass	~10	230	35	0.76	^17^
Bare SnO_2_	15 nm + 140 nm NPs	6.4	480	40	1.2	^15^
Bare SnO_2_	18 nm NPs	1.7	470	40	0.5	^22^
Bare SnO_2_	7.92 nm NPs	5.76	445	49	1.26	^19^
Bare SnO_2_	Hollow spheres (20 nm NPs)	6.40	390	34	0.86	^8^
	Hollow spheres (20 nm NPs) + TiCl4 treatment	14.59	765	54	6.02	^8^
Bare SnO_2_	6.5 nm NPs	6.1	292	37	0.66	^21^
Bare SnO_2_	18 nm NPs	5.7	320	27	0.51	^16^
	Hierarchical octahedral	6.84	543	56	2.07	^9^
	Hierarchical octahedral + TiO2 blocking layer	10.96	604	47.9	3.17	^9^
Bare SnO_2_	15 nm NPs	2.5	335	–	–	^14^
Commercial SnO_2_NPs	80–100 nm^‡^	6.71	330	47.96	1.07	^20^
SnO_2_ mesoporous microspheres	Microsphere sizes 1.2–1.5 μm (composed of packed nanobeads, 10–15 nm)	10.08	400	54.44	2.31	^20^
Bare SnO_2_	8–20 nm NPs	3.62	340	30.20	0.36	^23^

Open circuit photovoltage value observed in the present work is the highest recorded for SnO_2_-based DSSCs where neither TiO_2_ buffer layer is applied to the conductive substrate nor TiCl_4_ post treatment is used to cap the SnO_2_ ETM. This outcome is of extreme relevance: it indicates, indeed, that the synthesized SnO_2_ NPs feature an appropriate charge transport skill, with reduced exciton recombination, as compared with previously investigated photoanodes based on the same MOx.

**Figure 6:**
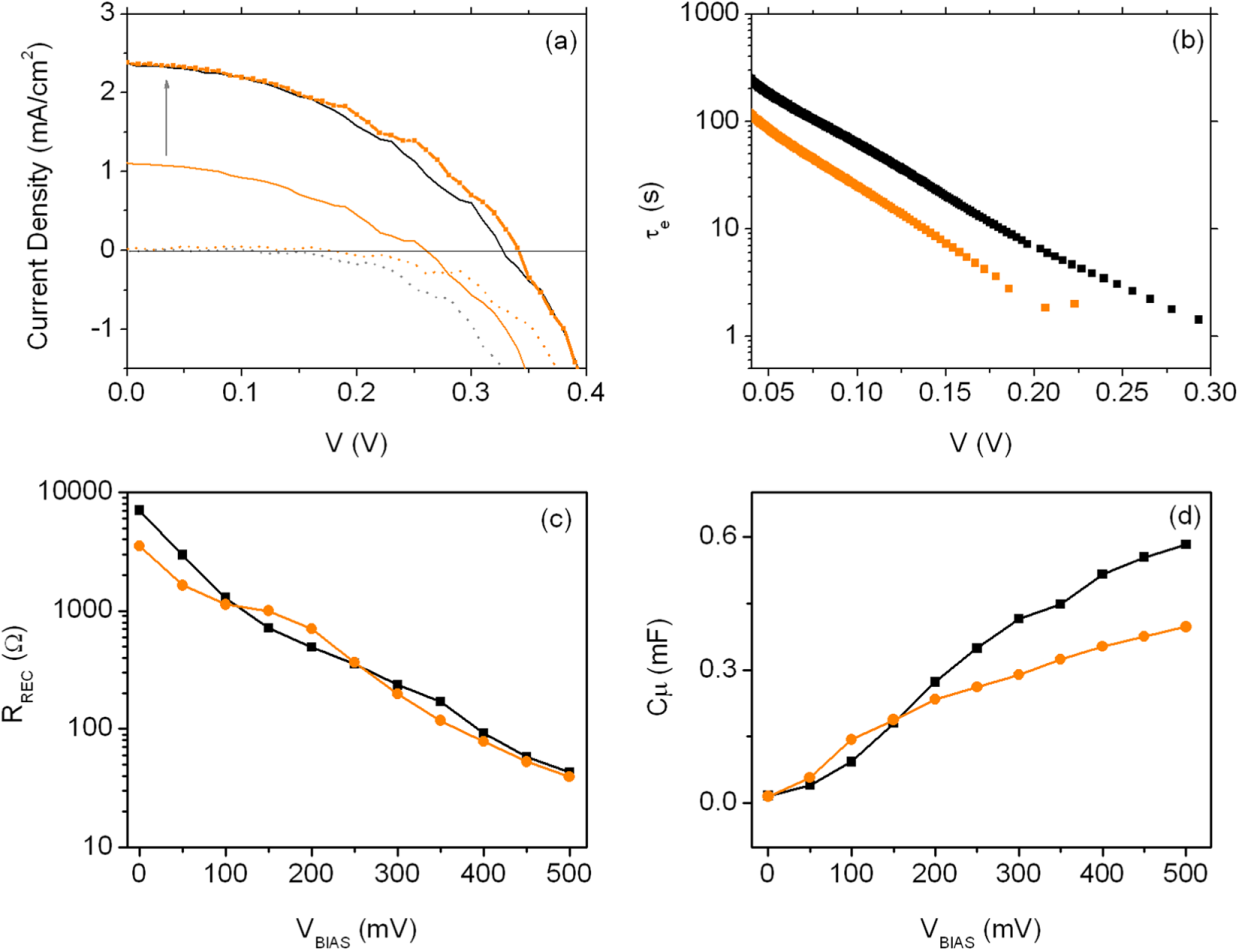
Comparison between DSSC sensitized with BI55 (orange line and markers) and BI54 (black line and markers). (**a**) J-V characteristics (solid lines: simulated solar light; dotted line: dark); (**b**): electron lifetime; (**c**) recombination resistance; (**d**) chemical capacitance.

When dye **BI54** is applied as light harvester (Fig. 6c,d), the observed open circuit photovoltage is still higher for TiO_2_ (540 mV) than for SnO_2_ (180 mV), for which, however, it is reduced beyond the expectations. On the contrary, short circuit current density is almost two times higher when SnO_2_ is applied as ETM (2.605 mA/cm^2^ *vs*. 1.376 mA/cm^2^ recorded for TiO_2_-based DSSC). One of the advantages of SnO_2_ over TiO_2_ is the higher electron mobility: mobility reported for SnO_2_ (μ_e_ ≈ 250 cm^2^V^−1^s^−1^ for SnO_2_ single crystal^53^ and μ_e_ ≈ 125 cm^2^V^−1^s^−1^ for SnO_2_ nanostructures^54^) is order of magnitude higher than the one reported for TiO_2_ (μ_e_ < 1 cm^2^V^−1^s^−1^ for TiO_2_ single crystal^55^). However, this higher mobility is of no help with Ru-based light harvesters, as widely shown in literature, while it is capable of spurring the photogenerated current when a light harvester with specifically designed feature is applied.

Electron lifetime appears slightly longer for the TiO_2_-based device (Fig. 6c). The Reader should however be aware that a longer electron lifetime may also be correlated to the presence of charges trapped in undesired surface states, which are of no use in converting the solar light.

Analysis of *J-V* characteristics in dark provides useful information on the origin of the seemingly inconsistent behavior of the two MOx. We should herein remind that V_*OC*_ should ideally match the difference in energy between the conduction band (CB) of the ETM and the standard redox potential of the electrolyte couple (the iodine/triiodine in the systems analyzed here). Such view well explains the difference in V_*OC*_ observed for TiO_2_- and SnO_2_-based DSSCs exploiting dye N719 as a dye (see the energy scheme reported in Fig. 2), but it is no longer satisfactory when **BI54** is instead used. *J-V* characteristics in dark (reported as dotted lines for both MOx and both dyes in Fig. 6a,c) show an increased dark current for SnO_2_-based devices, irrespectively of the used dye. The difference in dark current is moderate for devices sensitized with dye N719 (Fig. 6a): for both cells dark current onset is around 350 mV. This difference is however more pronounced in DSSCs sensitized with dye **BI54** (Fig. 6c), for which the onset moved below 200 mV. The dark current in solar cells comes from charge recombination: an enhancement of dark current is then associated to a faster recombination^22^,which is usually reflected in lower open circuit voltage values, as it is in the present cases. Given that the two devices have exactly the same structure but for the sensitizer, this effect is to be correlated with the nature of the ZnPc light harvester, which apparently is inhibiting the charge transport more than dye N719. The differences in the onset of dark current recorded for dye N719 and **BI54** can be ascribed to the skill of N719 in partially blocking the recombination at the FTO||electrolyte interface^56^, which is not featured by the ZnPc **BI54**. One of the reasons why SnO_2_ is investigated as alternative ETM to TiO_2_ in excitonic solar cells is, as mentioned, the higher electron mobility featured by this MOx. However, it should not be neglected that this higher electron mobility might also result in enhanced recombination, leading to a more pronounced electron-hole recombination.

The most relevant photovoltaic parameters of SnO_2_-based DSSCs sensitized with ZnPCs **BI55** or **BI54** are summarized in Fig. 6 (functional parameters are reported in Table 1, entries 5 and 6).

Interestingly the shapes of *J-V* characteristics are quite similar and suggest no difference between the resistances of the two devices (shunt resistance, R_SH_, and series resistance, R_S_). Since the values for V_*OC*_ only differ for 67 mV (260 mV and 327 mV for sensitizer **BI55** and sensitizer **BI54**, respectively), a different adsorption mode of the dyes to the surface of oxide might be responsible of the difference in term of J_*SC*_. The Fermi level E_F_ and the electric field that assists the injection of the charges are possibly similar for the two devices and the density of the charge is lower in the case of **BI55**.

Also in this case, dark *J-V* characteristics bring useful information to explain the difference in performances. The device sensitized with ZnPc **BI55** shows an enhanced dark current, which, accordingly to what mentioned above, suggests a reduced skill of the dye in blocking the recombination at the FTO||electrolyte interface. By equaling the short circuit currents^57^, i.e. the charge injection (orange markers in Fig. 6a), it is possible to observe that the DSSCs work in the same way. This strongly suggests that the limiting factor in the **BI55**-sensitized device is the charge injection from the LUMO to the SnO_2_ CB. The EIS analysis applied to these two cells confirms this hypothesis (Fig. 6c,d). The two devices show no differences in R_REC_ (Fig. 6c), while the trends of C_μ_ (Fig. 6d) suggests a downward of the CB for the oxide sensitized with BI54, as previously reported^46^. Indeed, the slope of C_μ_ is different when the two ZnPcs are applied: C_μ_ pertaining to the device sensitized with ZnPc **BI55** is less steep as compared with that observed in case of **BI54**, indicating a beneficial and better capping of the SnO_2_ trap states by this latter. We have reported similar observations when TiO_2_ is applied as ETM and comparison is made between the dyes N719 and **BI54**^46^. Also in that case, poor injection was found responsible for the lower functional performances.

## Conclusions

We presented a study on the use of two unsymmetrically substituted ZnPcs as light harvesters in DSSCs exploiting either TiO_2_ or SnO_2_ nanoparticles as ETM.

Investigated molecules have been especially designed to fulfil the requirements identified by computational models as for energy level alignment between their LUMO and the MOx CB, good charge injection and absorption range extended to the infrared portion of the solar spectrum.

On the other hand, SnO_2_ nanoparticles developed in the present study have shown very nice performances when the commercial Ru(II)-based dye N719 is applied as sensitizer as compared with previously reported tin dioxide photoanodes, featuring a photogenerated current density comparable with that observed with TiO_2_ photoandoes and very good open circuit photovoltage.

Despite the excellent preconditions for both components, device functional performances proved to be below the expectations (less than 1% as for photoconversion efficiency). Main reasons behind this outcome have been identified in the poor charge injection from the excited ZnPcs to the CB of SnO_2_ and low capability of ZnPcs in contributing to block the recombination at the FTO||electrolyte interface. As in the case of TiO_2_-based DSSCs, our findings also confirm the ease of aggregation of ZnPcs on the MOx surface its negative impact on the device performance. We will thus focus our efforts on improving the performance of SnO_2_-based devices through specific modifications of the substitution pattern of the phthalocyanine ring, according to a strategy recently demonstrated in the case of perovskite solar cells featuring ZnPcs as hole transporting materials^58^.

While hypotheses built up by molecular modelling are useful hints for the design of new photosensitizers, this work confirms that a thorough analysis of the results obtained through experimental tests remains crucial for further structural modifications and refinements. Indeed, many parameters play a relevant role in the working mechanism of a solar energy converting devices and unexpected factors may reveal more critical than expected.

## Methods

### Dye-sensitized solar cells

#### SnO_2_ nanoparticles synthesis

In a round bottomed flask 1.2 ml SnCl_4_ were dissolved in 100 ml of methanol. After the fumes had disappeared, 4 ml NH_4_OH (30% in water) were added dropwise in about 10 minutes. As soon as NH_4_OH was added, a white flocculate appeared in the reaction mixture. Mixture was let reacting for about 20 minutes at room temperature, and then the solvent was slowly evaporated at 80 °C in an oven (6–7 h). The raw product was then annealed at 450 °C for 2 h under air atmosphere. All chemicals were purchased from Sigma-Aldrich and used without any further purification.

#### SnO_2_ paste preparation

SnO_2_ paste was prepared by mixing 0.8 g of SnO_2_ nanoparticles powder with ethyl cellulose (0.7 g) and α-terpineol (1.5 ml), in ethanol/water dispersion medium (8/3.5, V/V). The mixture was kept under vigorous stirring overnight to obtain a homogenous paste suitable for tape casting.

#### Photoanode preparation

TiO_2_ pastes (Dyesol 20 nm anatase nanoparticles paste 18NR-T and WER2-O, composed of anatase nanoparticles with diameter between 150 and 250 nm) were purchased from Dyesol. The redox electrolyte was composed of 0.1 M LiI, 0.05 M I_2_, 0.6 M 1,2-dimethyl-3-*n*-propylimidazolium iodide, and 0.5 M 4-*tert*-butylpyridine dissolved in acetonitrile.

The TiO_2_ and SnO_2_ paste were deposited onto FTO glass (sheet resistance 10Ω /☐) by tape casting technique. The paste was dried 15 min at ambient conditions and then fired on hot plate for 6 min (125–130 °C) for the TiO_2_ based photoanodes and 10 min at ambient conditions and then fired on hot plate for 45 min (100 °C) for SnO_2_ based photoanodes. In the case of TiO_2_ paste two layers of transparent TiO_2_ (18NR-T) and one of scattering TiO_2_ (WER2-O) were deposited.

All the samples were finally annealed at 450 °C for 30 min under ambient atmosphere. Photoanode thickness was evaluated by stylus profilometry and found about 15 μm.

The sensitization of photoanodes was performed for 5 hours in dark condition using solutions containing 0.5 mM N719 in ethanol and 0.17 mM **BI54** or **BI55** in dichloromethane.

#### Device assembly

DSSCs were fabricated using dye sensitized oxide photoanodes and platinized FTO glass as a counter electrode (5 nm Pt thin film deposited by sputtering) with 60 μm-thick plastic spacers (Surlin from Solaronix). The I_3_^−^/I^−^ redox couple electrolyte was composed of 0.1 M LiI, 0.05 M I_2_, 0.6 M 1, 2-dimethyl-3-*n*-propylimidazolium iodide, and 0.5 M 4-*tert*-butylpyridine dissolved in acetonitrile.

Scanning electron microscopy analysis was carried out in a LEO 1525 FE-SEM.

Dye loading quantification and metal oxide surface area evaluation were performed by spectrophotometry, detaching the absorbed dye N719 by using a 0.1 M NaOH aqueous solution and recording the UV-Vis absorption spectra in a T80 spectrophotometer (PG Instruments) after building up a calibration curve with standard solutions; quartz cuvettes were used (1 cm optical path).

The current–voltage (I-V) characteristics of the fabricated cells were measured without masking by using a Keithley 2400 SourceMeter under simulated sunlight using an ABET2000 solar simulator at AM 1.5 G (100 mW cm^−2^) calibrated using a reference silicon cell. The electrochemical impedance spectroscopy (EIS) was carried out in dark conditions using a SOLARTRON 1260 A Impedance/Gain-Phase Analyzer, with an AC signal 20 mV in amplitude, in the frequency range between 100 mHz and 300 kHz. An external bias was applied from 0 to 100 mV above the V_*OC*_.

Electron lifetime of the photogenerated charges present in the CB of the dyed semiconductor is calculated from open circuit voltage decay using the following equation:^59^

$${\tau }_{n}=-\frac{{k}_{B}T}{e}{\left(\frac{d{V}_{OC}}{dt}\right)}^{-1}$$

where *k*_*B*_*T* is the thermal energy, e is the positive elementary charge.

## Supplementary information

Supplementary Information.

## References

[1] Hagfeldt A, Boschloo G, Sun L, Kloo L, Pettersson H (2010). Dye-sensitized solar cells. Chem. Rev..

[2] Shaikh JS (2018). Nanoarchitectures in dye-sensitized solar cells: metal oxides, oxide perovskites and carbon-based materials. Nanoscale.

[3] Concina I, Vomiero A (2015). Metal oxide semiconductors for dye- and quantum-dot-sensitized solar cells. Small.

[4] Zhuang S (2019). Cu modified ZnO nanoflowers as photoanode material for highly efficient dye sensitized solar cells. Electrochim. Acta.

[5] Xie F, Wang J, Li Y, Dou J, Wei M (2019). One-step synthesis of hierarchical SnO2/TiO2 composite hollow microspheres as an efficient scattering layer for dye-sensitized solar cells. Electrochim. Acta.

[6] Patil JV (2019). Influence of reduced graphene oxide-TiO2 composite nanofibers in organic indoline DN350 based due sensitized solar cells. Synth. Met..

[7] Shalini S (2016). Status and outlook of sensitizers/dyes used in dye sensitized solar cells (DSSC): a review. Int. J. Energy Res..

[8] Wang H (2012). SnO2 hollow nanospheres enclosed by single crystalline nanoparticles for highly efficient dye-sensitized solar cells. Cryst. Eng. Comm..

[9] Wang Y-F (2010). Hierarchical tin oxide octahedra for highly efficient dye-densitized solar cells. Chem. Eur. J..

[10] Elumalai NK, Jose R, Archana PS, Chellappan V, Ramakrishna S (2012). Charge transport through electrospun SnO2 Nanoflowers and nanofibers: role of surface trap density on electron transport dynamics. J. Phys. Chem. C.

[11] Shang G (2012). Facile synthesis of mesoporous tin oxide spheres and their applications in dye-sensitized solar cells. J. Phys. Chem. C.

[12] Song H (2013). A simple self-assembly route to single crystalline SnO2 nanorod growth by oriented attachment for dye sensitized solar cells. Nanoscale.

[13] Tarini M, Prakash N, Mathar Sahib IKM, Hayakawa Y (2017). Novel sugar apple-shaped SnO2 microspheres with light scattering effect in dye-sensitized solar cell application. IEEE J. Photolt..

[14] Tennakone, K., Kumara, G. R. R. A., Kottegoda I. R. M., Perera, V. P. S. An efficient dye-sensitized photoelectrochemical solar cell made from oxides of tin and zinc, *Chem. Commun.*, 15–16 (1999).

[15] Kay A, Grätzel M (2002). Dye-Sensitized Core-Shell Nanocrystals: Improved efficiency of mesoporous tin oxide electrodes coated with a thin layer of an insulating oxide. Chem. Mater..

[16] Chappel S, Chen S-G, Zaban A (2002). TiO2-coated nanoporous SnO2 electrodes for dye-sensitized solar cells. Langmuir.

[17] Prasittichai C, Hupp JT (2010). Surface modification of SnO2 photoelectrodes in dye-sensitized solar cells: significant improvements in photovoltage via Al2O3 atomic layer deposition. J. Phys. Chem. Lett..

[18] Desai UV, Xu C, Wu J, Gao D (2013). Hybrid TiO2−SnO2 nanotube arrays for dye-sensitized solar cells. J. Phys. Chem. C.

[19] Park N-G, Kang MG, Kim KM, Ryu KS, Chang SH (2004). Morphological and photoelectrochemical characterization of core-shell nanoparticle films for dye-sensitized solar cells: Zn-O type shell on SnO2 and TiO2 cores. Langmuir.

[20] Li Z (2013). Versatile nanobead-scaffolded N-SnO2 mesoporous microspheres: one-step synthesis and superb performance in dye-sensitized solar cell, gas sensor, and photocatalytic degradation of dye. J. Mater. Chem. A.

[21] Park N-G, Gu Kang M, Sun Ryu K, Man Kim K, Ho Chang S (2004). Photovoltaic characteristics of dye-sensitized surface-modified nanocrystalline SnO2 solar cells. J. Photochem. Photobiol. A: Chemistry.

[22] Green ANM, Palomares E, Haque SA, Kroon JM, Durrant JR (2005). Charge transport versus recombination in dye-sensitized solar cells employing nanocrystalline TiO2 and SnO2 films. J. Phys. Chem. B.

[23] Pereira MS, Lima FAS, Silva CB, Freire PTC, Vasconcelos IF (2017). Structural, morphological and optical properties of SnO2 nanoparticles obtained by a proteic sol–gel method and their application in dye-sensitized solar cells. J. Sol-Gel Sci. Technol..

[24] Urbani M, Ragoussi M-E, Nazeeruddin MK, Torres T (2019). Phthalocyanines for dye-sesitized solar cells. Coord. Chem. Rev..

[25] Wang A, Long L, Zhang C (2012). Synthesis of unsymmetrical phthalocyanines: a brief overview. Tetrahedron.

[26] Ragoussi M-E, Torres T (2014). Modern synthetic tools toward the preparation of sophisticated phthalocyanine-based photoactive systems. Chem. Asian J..

[27] Singh VK, Kanaparthi RK, Giribabu L (2014). Emerging molecular design strategies of unsymmetrical phthalocyanines for dye-sensitized solar cell applications. RSC Adv..

[28] Martín-Gomis L, Fernández-Lázaro F, Sastre-Santos Á (2014). Advances in phthalocyanine-sensitized solar cells (PcSSCs). J. Mater. Chem. A.

[29] Tejerina L, Martínez-Díaz MV, Nazeeruddin MK, Graetzel M, Torres T (2017). Role of the bulky aryloxy group at the non-peripheral position of phthalocyanines for dye sensitized solar cells. ChemPlusChem.

[30] Suanzes Pita J (2017). Pyridyl- and picolinic acid substituted zinc(II) phthalocyanines for dye-sensitized solar cells. ChemPlusChem.

[31] Martín-Gomis L, Parejo C, Alvarez JC, Fernández-Lázaro F, Sastre-Santos ÁL (2017). Dye sensitized solar cells (DSSCs) based on bulky tert-octylphenoxy-carboxyphenyl substituted phthalocyanine without the presence of co-adsorbents. Inorganica Chimica Acta.

[32] Kimura M (2017). Carbazole-fused zinc(II)-phthalocyanine sensitizers. Asian J. Org. Chem..

[33] Matsuzaki H (2014). Dye aggregation effect on interfacial electron-transfer dynamics in zinc phthalocyanine-sensitized solar cells. J. Phys. Chem. C.

[34] Zhang L, Cole JM (2017). Dye aggregation in dye-sensitized solar cells. J. Mater. Chem. A.

[35] Yum JH (2008). Effect of coadsorbent on the photovoltaic performance of zinc pthalocyanine-sensitized solar cells. Langmuir.

[36] Milan R (2015). ZnO@SnO2 engineered composite photoanodes for dye sensitized solar cells. Scientific Reports.

[37] Memarian N (2011). Hierarchically assembled ZnO nanocrystallites for high-efficiency dye-sensitized solar cells. Angew. Chem. Int. Ed..

[38] Selopal GS (2014). Effect of blocking layer to boost photoconversion efficiency in ZnO dye-sensitized solar cells. ACS Appl. Mater. Interfaces.

[39] Selopal GS (2016). Metal-free organic dyes for TiO2 and ZnO dye-sensitized solar cells. Scientific Reports.

[40] Pecnikaj I (2017). Fluorous molecules for dye-senzitized solar cells: synthesis and properties of di-branched, di-anchoring organic sensitizers containing fluorene subunits. New J. Chem..

[41] Tortelli S (2016). Property tuning in unsymmetrical alkoxy zinc phthalocyanines by introduction of perfluoro-tert-butoxy end groups. J. Fluorine Chem..

[42] Pozzi G (2013). Synthesis and photovoltaic applications of a 4,4′-spirobi[cyclopenta[2,1-b;3,4-b’]dithiophene]-bridged donor/acceptor Dye. Org. Lett..

[43] Yang L (2012). Theoretical design and screening of panchromatic phthalocyanine sensitizers derived from TT1 for dye-sensitized solar cells. J. Mol. Graphics Modell..

[44] Yang L (2012). Substituent effects on zinc phthalocyanine derivatives: a theoretical calculation and screening of sensitizer candidates for dye-sensitized solar cells. J. Mol. Graphics Modell..

[45] Linares-Flores C, Mendizabal F, Arratia-Pérez R, Inostroza N, Orellana C (2015). Substituents role in zinc phthalocyanine derivatives used as dye-sensitized solar cells. A theoretical study using Density Functional Theory. Chem. Phys. Lett..

[46] Milan R (2017). Dye-sensitized solar cells based on a push-pull zinc phthalocyanine bearing diphenylamine donor groups: computational predictions face experimental reality. Scientific Reports.

[47] Kimura M (2013). Molecular design rule of phthalocyanine dyes for highly efficient near-IR performance in dye-sensitized solar cells. Chem. Eur. J..

[48] Ragoussi ME (2014). Sterically hindered phthalocyanines for dye-sensitized solar cells: influence of the distance between the aromatic core and the anchoring group. Chem. Phys. Chem..

[49] Ikeuchi S, Agrawal M, Ezoe S, Mori S, Kimura M (2015). Enhanced Charge separation efficiency in pyridine-anchored phthalocyanine-sensitized solar cells by linker elongation. Chem. Asian J..

[50] Fonstad CG, Rediker RH (1971). Electrical properties of high quality stannic oxide crystals. J. Appl. Phys..

[51] Grätzel M (2001). Molecular photovoltaics that mimics photosynthesis. Pure Appl. Chem..

[52] Parks GA (1965). The isoelectric points of solid oxides, solid hydroxides, and aqueous hydroxo complex systems. Chem. Rev..

[53] Jarzebski M, Marton JP (1976). Physical properties of SnO_2_ materials. J. Electrochem. Soc..

[54] Arnold MS, Avouris P, Pan ZW, Wang ZL (2003). Field-effect transistors based on single semiconducting oxide nanobelts. J. Phys. Chem. B.

[55] Hendry E, Koeberg M, O’Regan B, Bonn M (2006). Local field effects on electron transport in nanostructured TiO_2_ revealed by terahertz spectroscopy. Nano Lett..

[56] Ito, S., *et al*. Control of dark current in photoelectrochemical (TiO2/I2–I32) and dye-sensitized solar cells, Chem. Commun. 4351–4353 (2005). 10.1039/b505718c 16113745

[57] Barea EM (2010). Energetic factors governing injection, regeneration and recombination in dye solar cells with phthalocyanine sensitizers. Energy Environ. Sci..

[58] Cho KT (2019). Perovskite solar cells: 18% efficiency using Zn(II) and Cu(II) octakis(diarylamine)phthalocyanines as hole-transporting materials. ACS Appl. Energy Mater..

[59] Zaban A, Greenshtein M, Bisquert J (2003). Determination of the electron lifetime in nanocrystalline dye solar cells by photovoltage decay measurements. Chem. Phys. Chem..

