# Supplementary material for: Zinc phthalocyanines as light harvesters for SnO2-based solar cells: a case study
Source: Sci Rep. 2020 Jan 24;10:1176. doi: 10.1038/s41598-020-58310-1 (PMC6981253; doi:10.1038/s41598-020-58310-1)
Supplement: Supplementary file 1 — Supplementary Information. [file 41598_2020_58310_MOESM1_ESM.pdf]

Supplementary Materials

Zinc phthalocyanines as light harvesters for SnO_2-based solar cells: a case study

Riccardo Milan[a,b], Gurpreet Singh Selopal[a,c,d], Marco Cavazzini[e], Simonetta Orlandi[e], Rita Boaretto[f], Stefano Caramori[f], Isabella Concina*[a,b,g], Gianluca Pozzi*[e]

[a] *Department of Information Engineering, University of Brescia, Via Valotti, 9 – 25131 Brescia, Italy*

[b] *CNR-INO SENSOR Laboratory, via Branze 45 – 25123 Brescia, Italy*

[c] *Institute of Fundamental and Frontier Sciences, University of Electronic Science and Technology of China, Chengdu 610054, P. R. China.*

[d] *Institut National de la Recherche Scientifique, Centre Énergie, Matériaux et Télécommunications, 1650 Boul. Lionel Boulet, Varennes QC J3X 1S2, Canada.*

[e] *Institute of Molecular Science and Technology, ISTM-CNR, Via Golgi 19, 20133 Milano, Italy*

[f] *Department of Chemical and Pharmaceutical Sciences, University of Ferrara, Via Borsari 46, 44121 Ferrara, Italy*

[g] *Division of Materials Science, Department of Engineering Sciences and Mathematics, Luleå University of Technology, 971 87 Luleå, Sweden*

* Corresponding authors e-mail address: isabella.concina@ltu.se; gianluca.pozzi@istm.cnr.it

Synthesis of ZnPc BI55

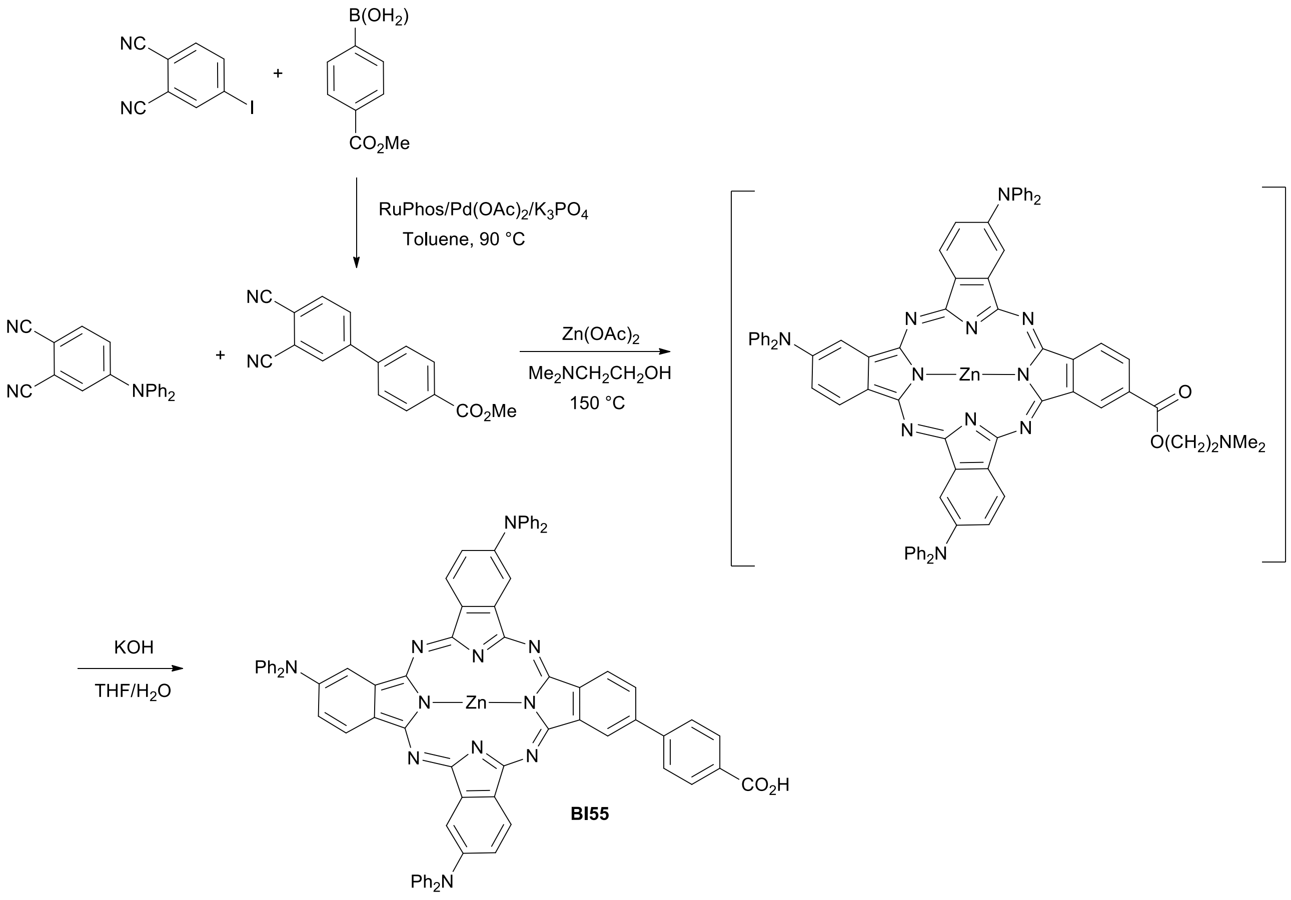

General remarks. Commercially available reagents were used as received. 4-(Diphenylamino)phthalonitrile and ZnPc **BI54** were synthesized as previously described. [1] Solvents were purified by standard methods and dried if necessary. Reactions were monitored by thin layer chromatography (TLC) that was conducted on plates precoated with silica gel Si 60-F254 (Merck, Germany). Column chromatography was conducted using silica gel Si 60, 0.063–0.200 mm (normal) or 0.040–0.063 mm (flash) (Merck, Darmstadt, Germany). UV-Vis measurements in solution were performed on a Nicolet Evolution 500 spectrophotometer (Thermo Electron Corporation). ^{1}H NMR and ^{13}C NMR spectra were recorded on a Bruker Avance 400 spectrometer; chemical shifts are indicated in parts per million downfield from $SiMe_4$, using the residual proton ($CHCl_3$ = 7.26 ppm) and carbon

($CDCl_3$ = 77.0 ppm) solvent resonances as the internal reference. Coupling constant values *J* are given in Hz. MALDI mass spectra were obtained with a TOF-TOF mass spectrometer Microflex LT (Bruker). Elemental analyses were carried out by the Departmental Service of Microanalysis (University of Milano).

4-(4-Methoxycarbonylphenyl)phthalonitrile. A flame dried Schlenk tube equipped with a stir bar was charged with 4-iodophthalonitrile (400 mg, 1.57 mmol), 4-(methoxycarbonylphenyl)boronic acid (380 mg 2.11 mmol), Ruphos (21 mg, 0.047 mmol), $Pd(OAc)_2$ (20 mg, 0.089 mmol) and potassium phosphate (580 mg, 2.76 mmol) under inert conditions. The Schlenk tube was evacuated and backfilled with nitrogen three times. Degassed toluene (4 mL) was added and the reactor was brought into an oil bath pre-heated at 90°C. The reaction mixure was stirred at this temperature for 20 h. After cooling to room temperature, the mixture was diluted with AcOEt, washed with H_2O, aqueous NaCl and dried over $MgSO_4$. The drying agent was removed by filtration and the solvent was evaporated in vacuum. The crude product was purified by flash column chromatograpy (silica gel, hexane/AcOEt 4/7) affording the title compound as a white solid (411 mg, 67%). ^1H NMR (400 MHz, $CDCl_3$) δ 8.19 (d, J = 8.4 Hz, 1H), 8.05 (d, J = 1.7 Hz, 1H), 7.97 (dd, J = 8.2, 1.8 Hz, 1H), 7.91 (d, J = 8.2 Hz, 1H), 7.66 (d, J = 8.4 Hz, 1H), 3.97 (s, 1H). ^{13}C NMR ($CDCl_3$, 100.61MHz) δ 165. 8, 145.4, 141.01, 134.1, 132.2, 131.7, 130.6, 130.2, 127.3, 116.7, 115.3, 115.2, 114.8, 61.5.

ZnPc ***BI55***. In a flame dried Schlenk tube equipped with a stir bar a mixture of 4-(4-methoxycarbonylphenyl)phthalonitrile (177 mg, 0.30 mmol), 4-(diphenylamino)phthalonitrile (79 mg, 0.60 mmol) and $Zn(OAc)_2 \cdot 2H_2O$ (36 mg, 0.16 mmol) was suspended in dimethylaminoethanol (12 mL) The mixture was stirred for 15 min under a nitrogen atmosphere and then heated at 145 °C for further 24 h. After cooling to room temperature the reaction mixture was poured into MeOH and the solid was collected by filtration on a Büchner funnel. The solid was thoroughly washed with MeOH, dried under

reduced pressure. This raw material was subjected to column chromatography (silica gel, THF). The product obtained (83 mg, mostly dimethylaminoethylic ester of BI51, MW = 1270.8 as determined by MALDI-TOF mass analysis) was suspended in THF (9 mL) and H_2O (3 mL). Powdered KOH (63 mg, 1.12 mmol) was added and the mixture was warmed to 70 °C and stirred for 20 h. The solvent was removed under reduced pressure and H_2O (10 mL) was added to the residue. The pH of the aqueous phase was adjusted to 3 by dropwise addition of concentrated HCl. The black solid formed was collected by filtration on a Hirsch funnel, washed with H_2O, air dried and purified by column chromatography (silica gel, THF). The title product **BI55** was obtained as a dark blue solid as a mixture of positional isomers. Yield: 25 mg (16.0%). UV-Vis (THF, 1.5 x 10^{-5} M): λ_{max}/nm (log ε) = 352 (5.00), 501 (4.42), 646 (4.62), 718 (5.22). Anal. Calcd for $C_{75}H_{47}N_{11}O_2Zn$: C 75.09, H 3.95, N 12.84. Found C 75.12, H 3.96, N 12.77. MALDI-TOF (DHB-THF): MW calcd. For $(C_{75}H_{47}N_{11}O_2Zn)^+$ 1197.3; found 1197,5.

Electrochemical characterization of BI55

The electrochemical behavior of **BI55** was investigated using cyclic voltammetric technique in THF solvent. The oxidation potentials were determined from half-wave potentials ($E_{1/2}$) (E_{ox}–E_{red})/2 by cyclic voltammetry . Electrochemical characterizations were performed through an Autolab PGSTAT30 (Ecochemie) potentiostat/galvanostat interfaced with a PC under GPES software. For cyclic voltammetry, a three electrode, one compartment electrochemical cell was used. The reference electrode was SCE (with saturated KCl solution); the auxiliary electrode was Pt and the working electrode was glassy carbon. The working electrode was polished subsequently with 1 and 0.3 mm alumina powder and then rinsed with distilled water. All experiments were carried out in THF (Aldrich) using 0.1 M tetrabutylammonium hexafluorophosphate ($TBAPF_6$, Fluka, for electrochemical analysis 99%). The electrolyte solution was degassed with nitrogen prior to measurements. All redox potentials were referenced against SCE. Peak potentials were measured at scan rates variable from 0.02 Vs^{-1} to 3 Vs^{-1}. The heterogeneous electron transfer kinetics at glassy carbon interfaces resulted in quasi-reversible, diffusion-limited waves, with chemical reversibility for lower potential (0.6 V vs. SCE) attained for scan speed > 100 mV/s

Figure S1. Electrochemical analyses of dye **BI55** (WE:glassy carbon, CE:Pt, RE:SCE).

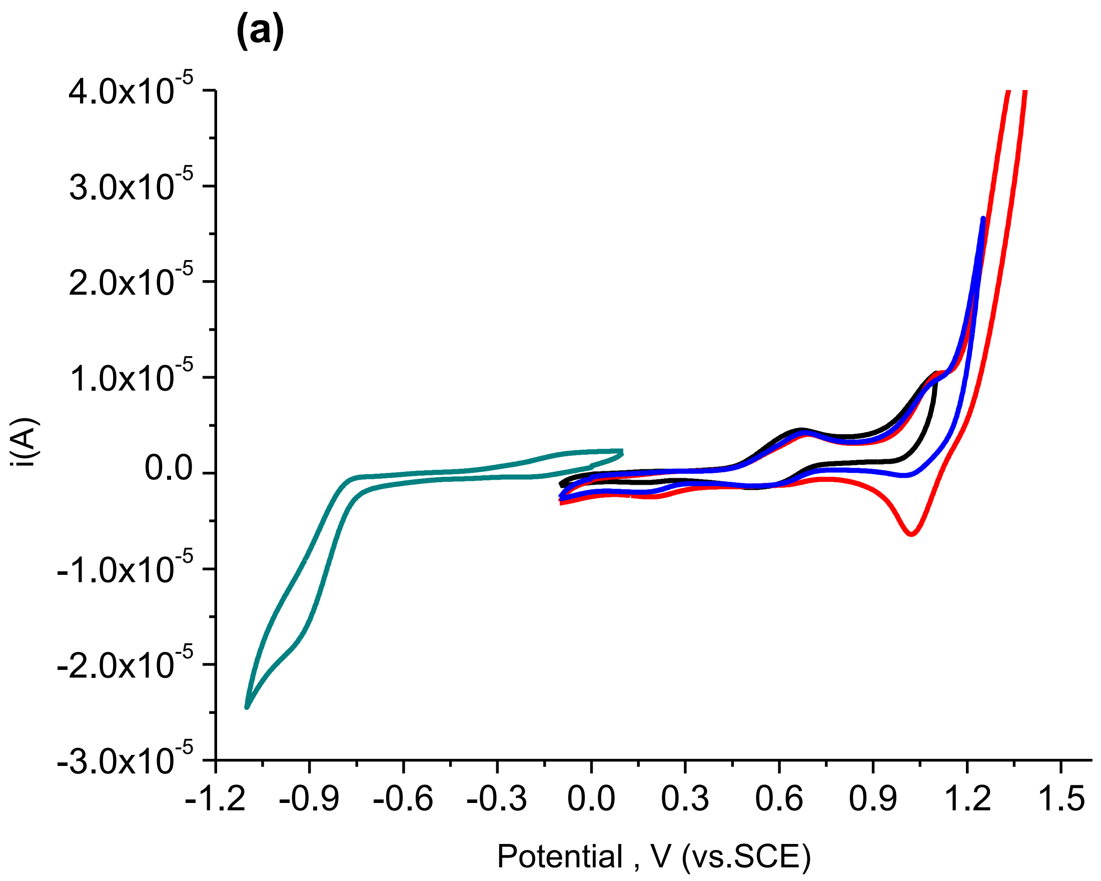

(a) Cyclic voltammetry recorded in THF at different potentials; scan rate 0.1 V/s.

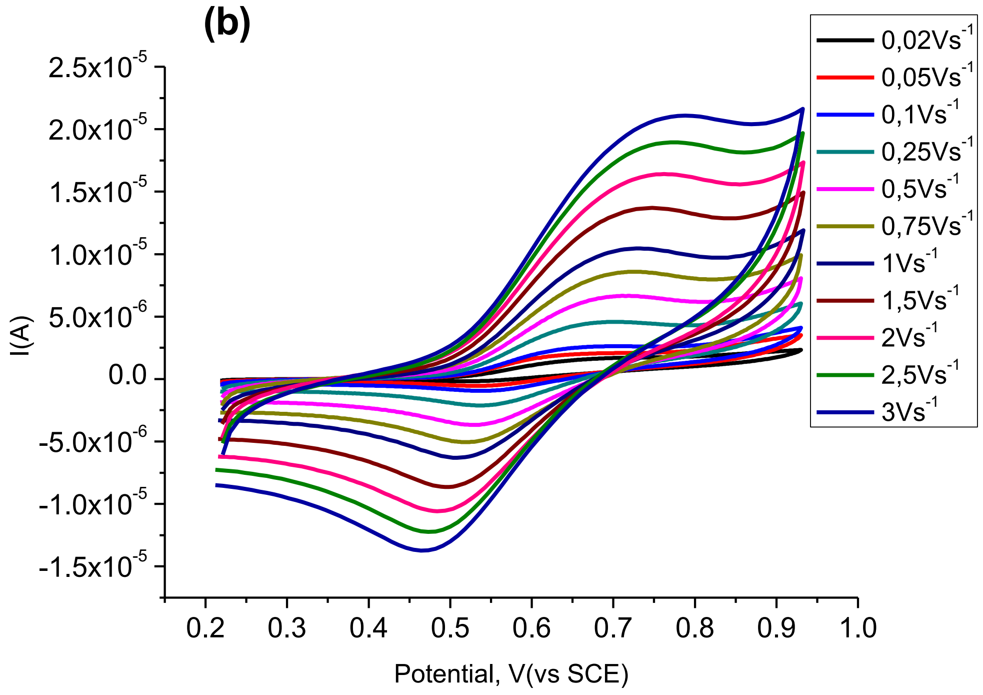

(b) Cyclic voltammetry recorded in THF at different scan rates (from 0.02 V/s to 3V/s).

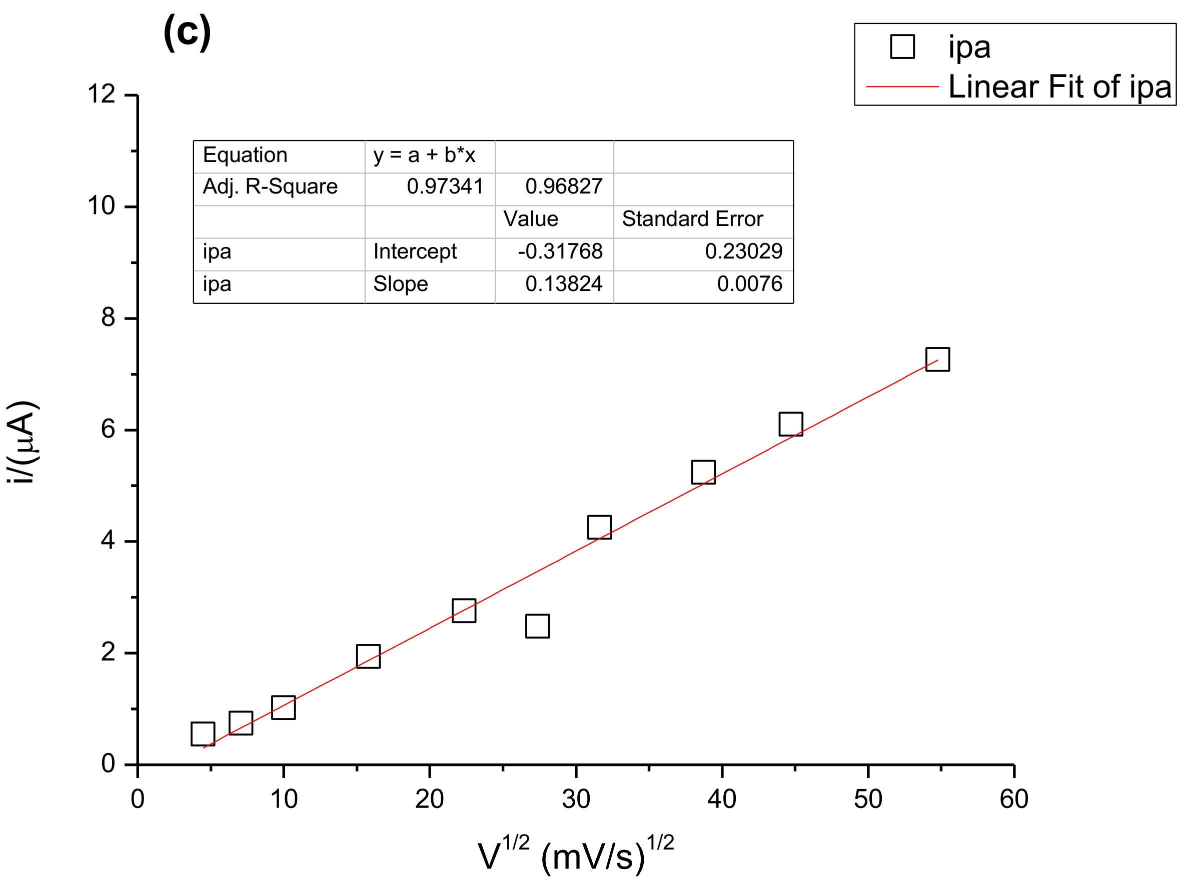

(c) Anodic peak current vs $\sqrt{v}$.

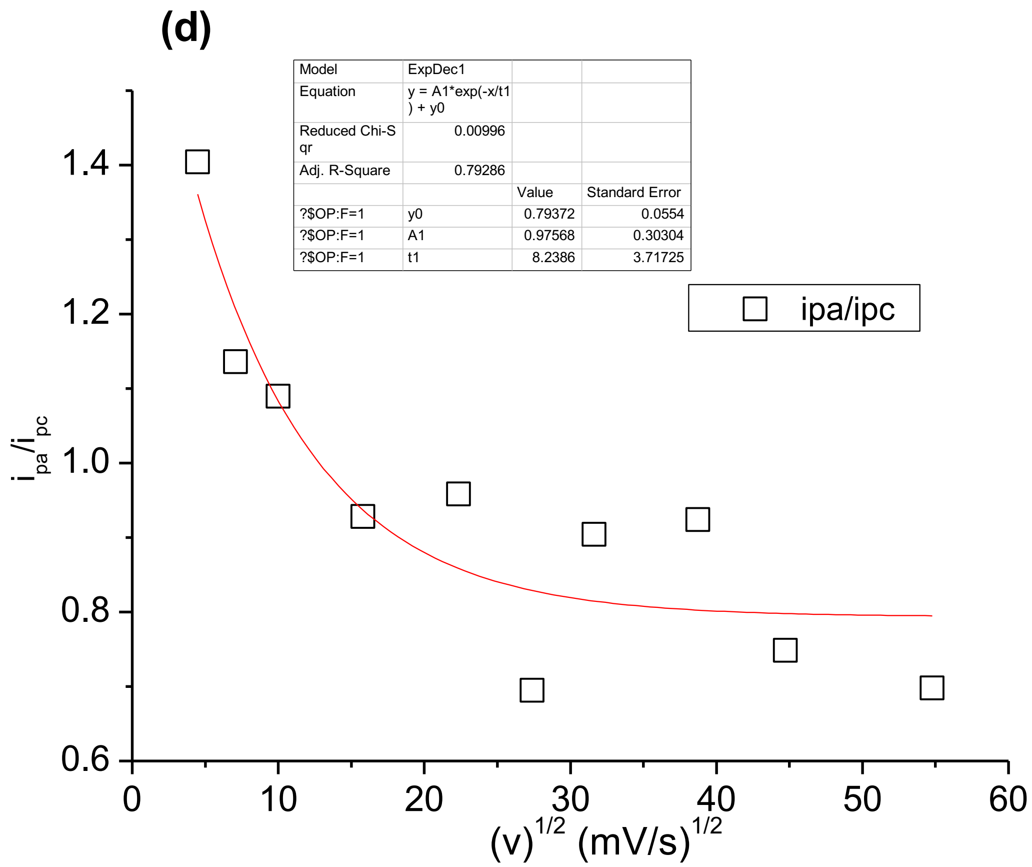

(d) Anodic vs Cathodic peak current ratio as a function of $\sqrt{v}$.

Table S1. Energy levels of **BI55** frontier molecular orbitals.

Dye	λ_{abs}(nm)	λem (nm)	E^{00}(eV)[a]	$E_{(HOMO)}$ vs NHE(V) (vs vacuum) (eV)[b]	$E_{(LUMO)}$ vs NHE (V) (vs vacuum) (eV)[c]
**BI 55**	498<br>646<br>718	733	1.71	1.05 (-5.55)	-0.66 (-3.84)

[a] Calculated from the intersection of normalized absorption and emission spectra. [b] Evaluated from oxidation potential obtained from CV, using -4.5 eV potential for NHE vs. vacuum. [c] Obtained from $E^{00} - E_{HOMO}$.

Figure S2 a shows the FTIR spectra of SnO_2 nanoparticles before and after thermal treatment. Before calcination several vibrations are observed, ascribable to the presence of residual methanol, together with bands attributed to SnOOH (1442 and 1408 cm^{-1}). [1] Lattice vibrations of SnO_2 expected below 800 cm^{-1} are detected with very low intensity (inset in Figure 3 a). Calcination process is effective in polishing SnO_2 from residuals: the IR spectrum shows indeed only contributions from adsorbed humidity (broad band 3500-2700 cm^{-1}) and the O-Sn-O and Sn-O stretching vibration below 800 cm^{-1} (inset in Figure 3 a). [2],[3]

The structure of SnO_2 influences the incident light reflection: the small NPs have lateral dimensions below the light wavelength, which result in a fair reflectance over a wide range as high as 60% (Figure S2 b).

Figure S2. (a) FTIR spectra of as prepared SnO_2 nanoparticles (red line) and SnO_2 nanoparticles after annealing (black line). (b) Reflectance spectrum of annealed SnO_2 nanoparticles.

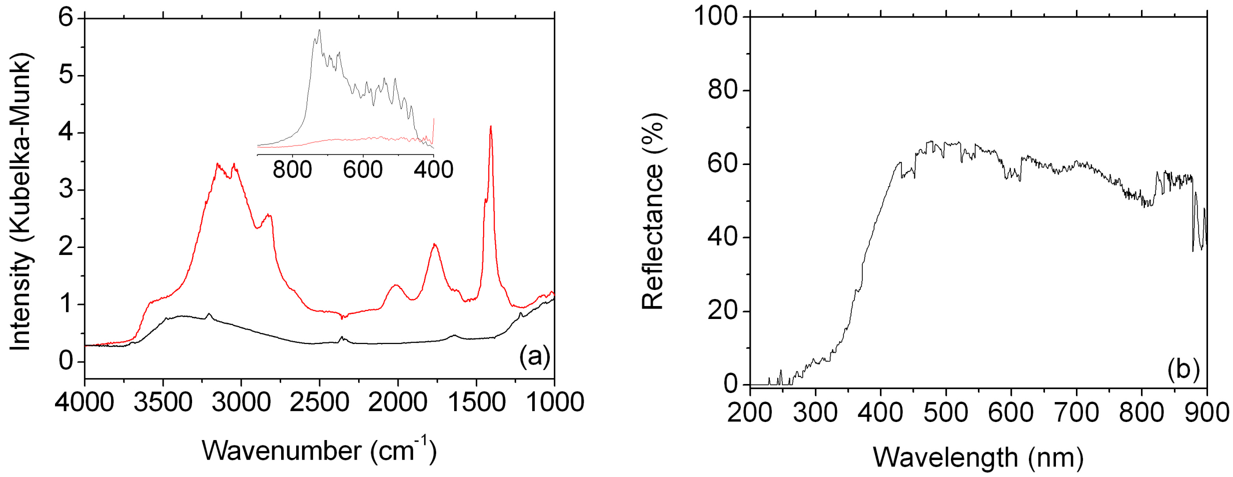

Figure S3. Comparison between DSSCs in which DCA was added (red line) or not added (black line) during the sensitization procedure with **BI54.**

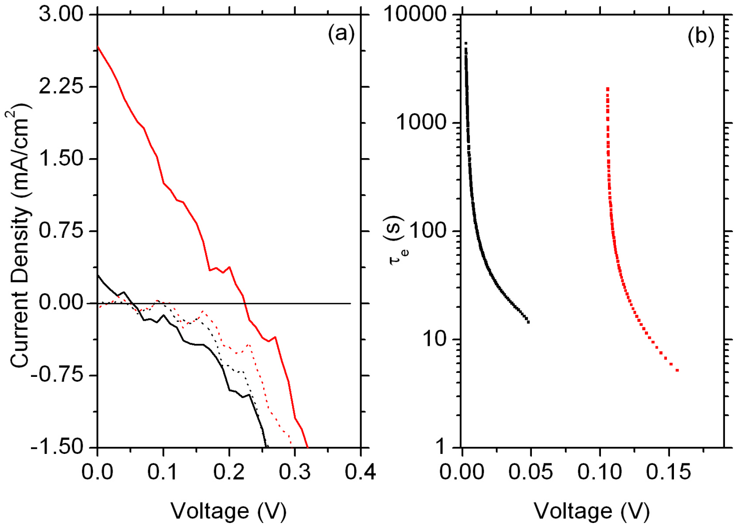

References

[1] Fujihara, S., Maeda, T., Ohgi, H., Hosono, E., Imai, H., Kim, S.-H. Hydrothermal Routes To Prepare Nanocrystalline Mesoporous SnO_2 Having High Thermal Stability, *Langmuir* **20**, 6476-6481 (2004).

[2] Ocala, M., Serna C.J., Matijevič, E. Formation of monodispersed SnO_2 powders of various morphologies, *Colloid. Polym. Sci.* **273**, 681-686 (1995).

[3] Orel, B., Lavrenčič-Štangar, U., Crnjak-Orel, Z., Bukovec P., Kosec, M. Structural and FTIR spectroscopic studies of gel-xerogel-oxide transitions of SnO_2 and SnO_2•Sb powders and dip-coated films prepared via inorganic sol-gel route, J*ournal of Non-Crystalline Solids* **167**, 272-288 (1994).

[4] R. Milan, G. S. Selopal, M. Cavazzini, S. Orlandi, R. Boaretto, S. Caramori, I. Concina, G. Pozzi, Sci. Rep. 2017, 7, 15675
